# *Clinopodium gracile* Alleviates Metabolic Dysfunction-Associated Steatotic Liver Disease by Upregulating Peroxisome Proliferator-Activated Receptor α and Inhibiting Mitochondrial Oxidative Damage

**DOI:** 10.3390/antiox13091136

**Published:** 2024-09-20

**Authors:** Mingshi Ren, Jiayue Ren, Jianmei Zheng, Xiaotong Sha, Yining Lin, Feihua Wu

**Affiliations:** School of Traditional Chinese Pharmacy, China Pharmaceutical University, Nanjing 211198, China; 18720033272@163.com (M.R.); jiayueren2017@163.com (J.R.); zjmei77@163.com (J.Z.); sxt08042@163.com (X.S.)

**Keywords:** *Clinopodium gracile*, metabolic dysfunction-associated steatotic liver disease, oxidative stress, lipid metabolism, mitochondrial damage

## Abstract

The most prevalent chronic liver disease, known as metabolic dysfunction-associated steatotic liver disease (MASLD), is characterized by an excessive accumulation of lipids and oxidative damage. *Clinopodium gracile*, a natural herbal medicine widely used by Chinese folk, has antioxidative, anti-inflammatory, and lipid metabolism-regulating effects. Here, we explored the effect of *C. gracile* extract (CGE) on MASLD using palmitic acid (PA)-induced hepatocytes and high-fat diet (HFD)-fed mice. In vitro, CGE could promote fatty acid oxidation and inhibit fatty acid synthesis and uptake to reduce lipid accumulation by regulating PPARα activation. Moreover, CGE could inhibit reactive oxygen species production and maintain mitochondrial homeostasis in PA-induced HepG2 cells. In vivo, animal study results indicated that CGE could effectively reduce lipid metabolism disorder, inhibit oxidative stress, and upregulate PPARα protein in the liver of HFD-fed mice. Molecular docking results also showed that active compounds isolated from CGE had low binding energy and highly stable binding with PPARα. In summary, these findings reveal that CGE may be a potential therapeutic candidate for MASLD and act by upregulating PPARα to reduce lipid accumulation and suppress mitochondrial oxidative damage.

## 1. Introduction

Metabolic dysfunction-associated steatotic liver disease (MASLD), previously referred to as non-alcoholic fatty liver disease, is a common chronic hepatic disorder worldwide. The MASLD definition highlights the importance of metabolic factors in steatotic liver disease. Fatty liver patients with obesity, overweight, insulin resistance, dyslipidemia, and other metabolic factors can be diagnosed as MASLD [1]. The estimated prevalence of MASLD ranges from 25% to 34%, with a subset of 2–5% progressing to metabolic dysfunction-associated steatohepatitis (MASH) in the general population. The pathogenesis of MASLD involves the excessive accumulation of lipids in hepatocytes and hepatic inflammation and fibrosis. Long-term exposure of the liver to excessive lipids results in the disorder of lipid metabolism, such as the excessive uptake of exogenous fats and increased production of endogenous fats, as well as the weakened ability to lipolysis [2]. At this time, the pathways related to the synthesis of fat are abnormally activated, insulin resistance occurs, and free fatty acids continue to accumulate in the liver [3]. Excessive free fatty acids and their metabolites produce lipotoxicity, which induces hepatic oxidative stress, endoplasmic reticulum stress, mitochondrial damage, and inflammation, thereby promoting the progression of hepatic steatosis to MASH [4].

Hepatic lipid accumulation is a crucial trigger for the development of MASLD. Lipotoxicity can result in hepatic injury through various mechanisms, such as oxidative stress, inflammation, and the activation of fibrogenic pathways [5]. Peroxisome proliferator-activated receptors (PPARs) regulate the metabolic homeostasis of fatty acids in various tissues, including the liver, adipose tissue, muscle, and other organs [6]. The predominant expression of PPARα occurs in hepatic tissue, where it governs the regulation of fatty acids synthesis, translocation, and β-oxidation. Firstly, the regulation of carnitine palmitoyltransferase 1A (CPT1A), a crucial regulator of fatty acid oxidation (FAO), is regulated by PPARα [7]. The mitochondria serve as the primary sites for FAO. Within the cytoplasm, fatty acids undergo activation to form acyl-CoA, which is subsequently transported into the mitochondrial matrix via CAT1A-mediated translocation across the mitochondrial membrane for subsequent FAO [8]. Secondly, the activation of PPARα can effectively attenuate the hepatic synthesis of fatty acids and accumulation of triglycerides (TGs) by reducing sterol-regulatory element-binding protein (SREBP-1c) expression [9,10]. PPARα regulates the expression of fatty acid synthesis genes, such as fatty acid synthase (FASN) and acetyl-CoA carboxylase 1 (ACC1), which are intricately associated with SREBP-1c [11]. Furthermore, PPARα specifically regulates the cluster of differentiation 36 (CD36) to maintain the intracellular homeostasis of fatty acids [12,13]. It has been reported that primary human hepatocytes treated with the PPARα ligand (GW7647) express a series of differential genes whose functional annotations are enriched in lipid metabolism, transport, and synthesis. PPARα regulates the expression of downstream lipid metabolism-related genes through direct or indirect genomic binding [14]. Therefore, PPARα is expected to be a potential target for the treatment of MASLD.

The mitochondria, which are highly concentrated in hepatocytes, serve as the primary site for lipid metabolism. Reactive oxygen species (ROS), predominantly generated within intracellular mitochondria, govern the oxidative stress state. The dysregulation of lipid metabolism results in disruptions in the mitochondrial electron transport chain, thereby resulting in an excessive production of ROS [15,16,17]. Normally, oxygen-free radicals generated during the mitochondrial electron transport chain are scavenged by glutathione peroxidase (GSH-Px) and superoxide dismutase (SOD). However, excessive ROS produced in hepatocytes exceeds the clearance capacity of antioxidant enzymes and causes continuous oxidative stress in MASLD mice [18,19]. Excessive ROS generation makes mitochondrial membrane potential decrease, mitochondrial membrane permeability increase, and cytochrome C (Cyt.c) release increase, resulting in mitochondrial oxidative damage [20]. During MASLD progresses to MASH, there is an obvious impairment of mitochondrial function in hepatocytes, characterized by alterations in mitochondrial structure, damage to mitochondrial DNA, abnormalities in fatty acid oxidation, and the excessive generation of ROS [21,22]. Therefore, reducing the production of ROS and inhibiting the oxidative damage of mitochondria may be an effective means to alleviate MASLD.

The traditional Chinese herb *Clinopodium gracile* belongs to the Lamiaceae family, which encompasses approximately 20 species of *Clinopodium* worldwide [23]. *Clinopodium chinense* (Benth.) O. Ktze and *C. gracile* belong to the same cluster in the pharmacognosy classification. Recent studies have demonstrated the pharmacological efficacy of *C. chinense* (Benth.) O. Ktze, including its hypoglycemic effect, cholesterol-lowering effect, cardiovascular protection, antioxidative and anti-inflammatory properties, as well as insulin resistance reduction [24,25,26,27]. The entire plant of *C*. *gracile* has the efficacy of clearing heat, detoxifying, reducing swelling, and relieving pain. Flavonoids, caffeic acid oligomers, and triterpene saponins are the main constituents of this plant. Local inhabitants of Fujian province in China commonly use *C. gracile* to treat diabetes and improve glucose and lipid metabolism disorders. However, the possible effect and the molecular mechanisms of *C. gracile* against hepatic steatosis are still unclear. Therefore, in this study, we investigated the effects of *C. gracile* extract (CGE) on hepatocyte lipid accumulation and the mitochondrial oxidative damage induced by palmitic acid (PA), as well as its ameliorating effect on high-fat diet (HFD)-fed MASLD mice.

## 2. Materials and Methods

### 2.1. Reagents

PA was purchased from Sinopharm Chemical Reagent Co., Ltd. (Shanghai, China). Fenofibrate was purchased from Solarbio Science & Technology Co., Ltd. (Beijing, China). Antibodies for PPARα were purchased from WanLei Biology (Shenyang, China). Antibodies against SREBP-1c were purchased from Santa Cruz Biotechnology (Dallas, TX, USA). Antibodies against GAPDH were purchased from Bioworld Technology Co., Ltd. (St. Louis Park, MN, USA). Antibodies for CPT1A and FASN were purchased from Proteintech (Wuhan, China). The total cholesterol (TC) and TG biochemical kits were obtained from Zhe Jiang Dong’ou Diagnostic Products (Wenzhou, China). Kits of SOD, GSH-Px, malondialdehyde (MDA), alanine aminotransferase (ALT), and aspartate aminotransferase (AST) were purchased from Nanjing Jiancheng Bioengineering Institute (Nanjing, China). The ROS assay kit was bought from Beyotime Biotechnology Co., Ltd. (Shanghai, China). The JC-1 mitochondrial membrane potential assay kit was purchased from MedChemExpress (Shanghai, China). The reference substances apigenin, narirutin, naringenin, didymin, hesperidin, and isosakuranetin were purchased from Shanxi Huike Botanical Development Co., Ltd. (Xian, China).

### 2.2. Plant Materials 

*C. gracile* herba was collected from Putian City, Fujian province, China. This was identified by Sheban Pu (Associated Professor, Department of Pharmacognosy, China Pharmaceutical University). The voucher specimen (CPU-CG20190825) was deposited in the Departments of Pharmacology of Chinese Materia Medica at China Pharmaceutical University.

### 2.3. Preparation of CGE Extract 

The whole plant (1.5 kg) of *C. gracile* was dried, crushed, and then extracted with 80% ethanol under reflux. The concentrated extract was successively extracted 4 times with petroleum ether and ethyl acetate, respectively. The ethyl acetate fractions were separated by macroporous resin D101 and eluted by 10%, 30%, 50%, 70%, and 90% ethyl alcohol solutions in turn. Finally, the 30~70% dry fractions were collected to obtain the CGE extract (14.1 g).

### 2.4. High-Performance Liquid Chromatography (HPLC) Analysis

Perform chromatographic analysis using an Agilent 1100 System (Agilent Technologies Inc., CA, USA) achieved liquid chromatographic separation with a C18 column (250 mm × 4.6 mm, 5 μm). The mobile phase consisted of HPLC-grade water (with 0.2% formic acid) as eluent A and acetonitrile as eluent B. The gradient elution process was set as follows: 0–10 min (20–30% B), 10–20 min (30–55% B), 20–30 min (55–65% B), 30–30.1 min (65–20% B), and 30.1–40 min (20% B). The flow rate of the mobile phase was 1.0 mL/min. An appropriate concentration solution of the above 6 standard substances and CGE were prepared with methanol, respectively. Each of the solutions was filtered using a 0.22 μm membrane filter, and then 10 μL was injected.

### 2.5. Cell Culture 

HepG2 cells were bought from the American Type Culture Collection (Manassas, VA, USA). The primary hepatocytes were isolated from male C57BL/6 mice using collagenase perfusion, as described previously [22]. The primary hepatocytes were cultured in Williams Medium E with 6% fetal bovine serum added. The HepG2 cells were cultured in Dulbecco’s modified Eagle’s medium (DMEM) with 10% fetal bovine serum added at 37 °C under a 5% CO_2_ atmosphere. The primary hepatocytes and HepG2 cells were treated with CGE (7.5, 15, and 30 μg/mL), fenofibrate (FNB, 100 μM), and PA (250 μM) for 24 h. For the preparation of a 250 μM PA solution, briefly, PA was added to 0.1 M NaOH solution, heated to 75 °C, and dissolved for 30 min to obtain a 20 mM PA saponification solution. Then, the PA saponification solution and 20% bovine serum albumin solution were mixed 1:1 to obtain a 10 mM PA solution, which was diluted 40 times with a medium before use. CGE was dissolved in dimethyl sulfoxide (DMSO) to a concentration of 30 mg/mL. It was diluted in DMEM before each experiment. The final DMSO concentration did not exceed 0.1% DMSO in the medium throughout the study. The groups in all experiments were consistently treated with 0.1% DMSO.

### 2.6. MTT Assay for the Viability of HepG2

The hepatocytes (8 × 10^3^ cells/well) were seeded in 96-well plates and kept in a 5% CO_2_ incubator at 37 °C. Then, the cells were treated with CGE (7.5, 15, 30, 40, 50, and 60 μg/mL) or FNB (50, 100, 150, and 200 μM) for a period of 24 h. After that, each well was supplemented with an MTT solution and incubated for an additional 4 h at 37 °C. Next, the MTT solution was discarded, and DMSO (150 μL) was added to each well. Finally, the absorbance was measured at a wavelength of 490nm using a Varioskan microplate reader (Thermo Fisher Scientific, Waltham, MA, USA).

### 2.7. Lipid Accumulation Assay

HepG2 cells (1 × 10^5^ cells/well) were seeded in 6-well plates and kept in a 5% CO_2_ incubator at 37 °C. The TG and TC content in HepG2 cells were assessed using biochemical kits followed by the instructions. Cells were removed from the incubator, washed with a phosphate buffer (PBS), and fixed for 15 min. The cells were then stained with oil red O for 1 h and restained with hematoxylin for another 15 s. The cells were ultimately examined through an Olympus microscope (Tokyo, Japan).

### 2.8. SOD, GSH-Px, MDA, and ATP Content Assay 

Cultured cells were collected and washed 2~3 times with PBS, sonication on ice, and then centrifuged for 10 min (4 °C 12,000× *g*/min). The supernatant was collected and assessed using biochemical kits followed by the instructions of the SOD, MDA, GSH-Px, and ATP kits.

### 2.9. ROS Production Assay

According to the instructions of the commercial kit, 1 mL of a serum-free medium containing the DCFH-DA probe (2 μM) was added to each well of cells and incubated in a constant temperature incubator for 30 min at 37 °C. After performing three washes with serum-free medium to eliminate an excess probe, the cells were examined using an inverted fluorescence microscope (Olympus, Tokyo, Japan).

### 2.10. Mitochondrial Membrane Potential Detection

Following the instructions of the commercial kit with the JC-1 probe, cells were washed in serum-free DMEM 3 times, with a 1 mL diluted JC-1 probe added to each well and incubated in an incubator for 20 min. After washing 3 times with PBS, 500 μL of serum-free medium was added to each well and observed with an inverted fluorescence microscope.

### 2.11. Transfection

To knock down PPARα, HepG2 cells were transfected with siRNA targeting PPARα (GenePharma Co., Ltd., Shanghai, China) using the lipofectamine™ 2000 transfection reagent (ThermoFisher, Waltham, MA, USA). Then, the transfection efficiency of cells was detected by Western blot.

### 2.12. Animal Treatment

C57BL/6 mice (male, 18~22 g body weight, 6~8 weeks old) were provided by the Comparative Medicine Centre of Yangzhou University (Yangzhou, China). Before the animal experiment, the mice were adaptively fed for three days in a specific pathogen-free animal room (23 ± 2 °C; a 12 h/12 h light–dark cycle). A total of 50 mice were divided into 5 groups, with 10 mice in each group. They are the normal group (Normal diet, ND), HFD group, HFD + CGE 40 mg/kg group, HFD + CGE 80 mg/kg group, and HFD + FNB 80 mg/kg group. A high-fat diet includes 60% fat + 19.4% protein + 20.6% carbohydrate + 1% cholesterol + 0.25% bile salts (TP23300-X, purchased from Nantong Trophy Company, Nantong, China). The mice were subjected to an HFD for a duration of 10 weeks, while the administration of CGE and FNB commenced from week 3 until week 10. After fasting for 12 h and drinking water freely, blood was collected from the abdominal aorta, centrifuged at 4 °C, 12,000× *g*/min for 10 min, and serum was collected before sacrifice. After weighing the liver, a part of the liver tissue was made into a 10% homogenate with isopropanol.

### 2.13. Biochemical Indices and Liver Function Assays 

The content of TC, TG, AST, ALT, SOD, MDA, and GSH-Px in serum was detected according to the commercial kit’s instructions. The TG and TC content in the 10% liver homogenate were also detected by biochemical kits.

### 2.14. Histopathology Analysis

Liver tissues were fixed in 4% formaldehyde for one night, and tissue sections were prepared by the PDX (Pharmacodynamic Evaluation Platform of China Pharmaceutical University). Sections were stained using hematoxylin-eosin (H&E) and oil red O staining according to standard procedures. Images were captured using a light microscope. 

### 2.15. Western Blot Analysis

The hepatic tissues and HepG2 cell samples were lysed using a lysis buffer solution. A 20 μg protein sample was used for electrophoresis, transmembrane, and blocking. Primary antibodies (1:1000 dilution) were added and incubated for 12 h at 4 °C. The next day, secondary antibodies (1:5000 dilution) were added and incubated for 2 h at room temperature. Protein immunoblotting was analyzed using an electrochemiluminescence (ECL) detection system. ImageJ version 1.8.0 software was used to analyze the gray values of each strip.

### 2.16. Molecular Docking Verification

To analyze the binding energy and interaction mode between the active compounds and PPARα target, AutoDockTools-1.5.7, protein–ligand docking software, was employed. The molecular structures of narirutin, hesperidin, rosmarinic acid, didymin, apigenin, isosakuranetin, and fenofibrate were retrieved from the PubChem website accessed on 3 August 2024 (https://pubchem.ncbi.nlm.nih.gov/). The 3D structure of PPARα (PDB ID, 1I7G; resolution, 2.20 Å) was downloaded from the PDB website accessed on 3 August 2024 (http://www.rcsb.org/pdb/home/home.do). Molecular docking studies were performed by AutoDockTools-1.5.7 accessed on 3 August 2024 (http://autodock.scripps.edu/).

### 2.17. Statistical Analysis

All data results were presented as the mean ± SEM, and statistical processing was performed by SPSS 21.0 (SPSS, Chicago, IL, USA). An independent sample *t*-test was used for data comparisons between the two groups. One-way analysis of variance (ANOVA) was used to compare the data of three or more groups. Tukey’s multiple comparison was used for post hoc tests. *p* values less than 0.05 were considered statistically significant.

## 3. Results

### 3.1. Characterization of the CGE by HPLC

**Figure 1:**
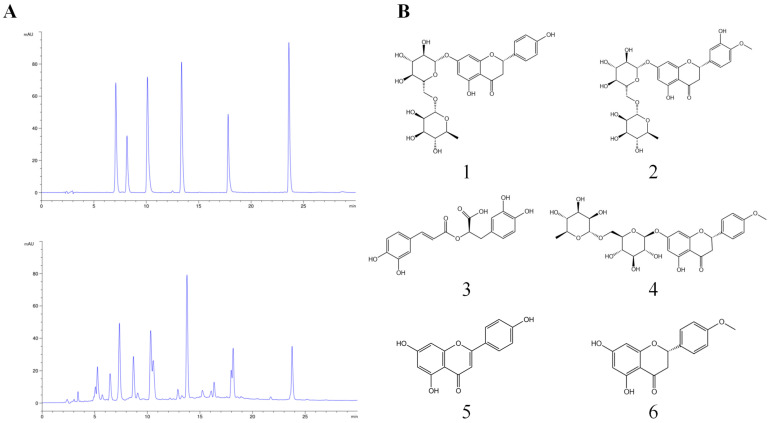
Analysis of chemical components in CCE using HPLC. (**A**) A mixture of reference compounds and HPLC chromatograms of CGE were detected at 280 nm. (**B**) The chemical structures of six components. 1: narirutin, 2: hesperidin, 3: rosmarinic acid, 4: didymin, 5: apigenin, and 6: isosakuranetin.

The extracts from *C. gracile* were analyzed by HPLC. A total of six ingredients were identified in CGE, including narirutin (Peak 1), hesperidin (Peak 2), rosmarinic acid (Peak 3), didymin (Peak 4), apigenin (Peak 5), and isosakuranetin (Peak 6). Among these compounds, the content of narirutin, hesperidin, didymin, and isosakuranetin in CGE were estimated to be 48.35, 80.36, 115.67, and 11.58 mg/g, respectively (Figure 1A,B).

### 3.2. CGE-Reduced Lipid Accumulation in PA-Treated Hepatocytes

**Figure 2:**
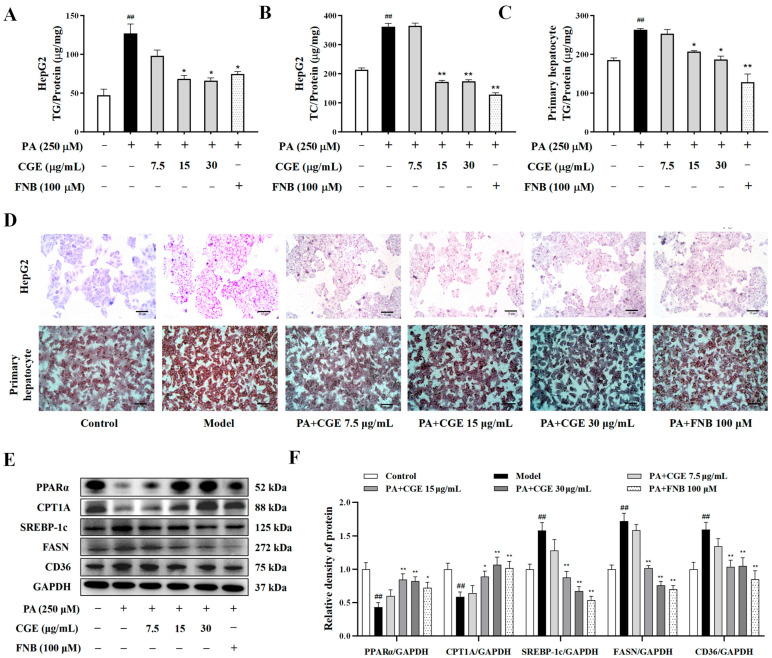
CGE reduced lipid accumulation in PA-treated hepatocytes. (**A**,**B**) The TG and TC contents of HepG2 cells were measured by a microplate reader. (**C**) the TG content was measured in primary hepatocytes. (**D**) Oil red O staining of primary hepatocytes and HepG2 cells, scale bar = 10 µm. (**E**,**F**) The expressions of the PPARα, CPT1A, SREBP-1c, FASN, and CD36 protein were determined by Western blot. Data are expressed as the mean ± SEM (*n* = 3), ^##^ *p* < 0.01 versus the untreated group; * *p* < 0.05, ** *p* < 0.01 versus the PA-only treated group.

We first examined the effects of different concentrations of CGE on the activity of HepG2 cells. CGE (7.5~50 μg/mL) had no obvious cytotoxicity (Supplementary Figure S1A), and FNB (0~200 μM) also had no significant effect on HepG2 cell viability (Supplementary Figure S1B). The excessive accumulation of intracellular TG is a key indicator of MASLD. Both CGE and FNB treatment reduced the content of intracellular TG and TC to different degrees in the HepG2 cell or primary hepatocytes (Figure 2A–C). Moreover, oil red O staining results showed that CGE treatment reduced intracellular lipid droplets in HepG2 cells and primary hepatocytes compared to the model group (Figure 2D). The expression of PPARα and CPT1A were decreased, and the expressions of SREBP-1c, FASN, and CD36 increased significantly in PA-induced HepG2 cells (Figure 2E,F). CGE treatment significantly reversed the PA-induced decrease in PPARα and CPT1A levels and the increase in SREBP-1c, FASN, and CD36 expression. These results demonstrated that CGE promoted lipid metabolism and reduced lipid accumulation in hepatocytes effectively.

### 3.3. CGE Attenuated Oxidative Stress in PA-Treated HepG2 Cells

**Figure 3:**
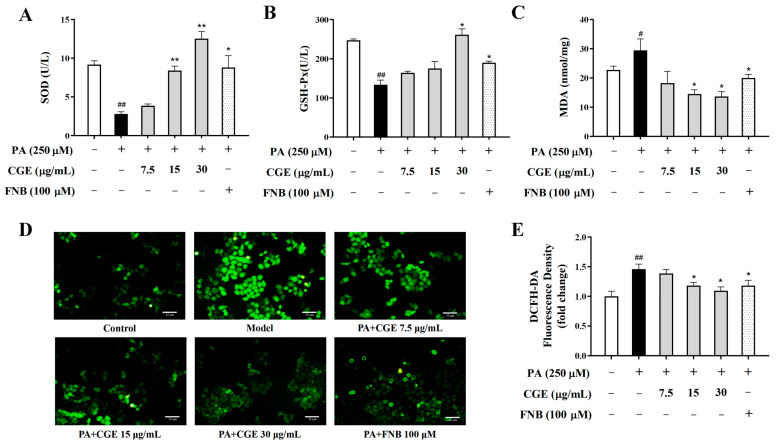
CGE attenuated oxidative stress in PA-treated HepG2 cells. (**A**–**C**) SOD, GSH-Px, and MDA were measured by a microplate reader. (**D**) The intracellular ROS level was measured by a fluorescence microscope, scale bar = 10 µm. (**E**) The relative green fluorescence intensity of DCFH-DA staining. Data are expressed as the mean ± SEM (*n* = 3), ^#^ *p* < 0.05, ^##^ *p* < 0.01 versus the untreated group; * *p* < 0.05, ** *p* < 0.01 versus the PA-only treated group.

Normally, hepatocytes resist oxidative stress by maintaining homeostasis of the intracellular antioxidant enzyme system. PA treatment decreased the SOD and GSH-Px activities while increasing the MDA content (Figure 3A–C). The intracellular ROS content was significantly increased in the model group (Figure 3D,E). Nevertheless, after CGE treatment, the SOD and GSH-Px activities were increased, and MDA and ROS content decreased significantly. The above results indicate that CGE attenuated oxidative stress in HepG2 cells treated with PA.

### 3.4. CGE Maintained Mitochondrial Homeostasis in PA-Treated HepG2 Cells

**Figure 4:**
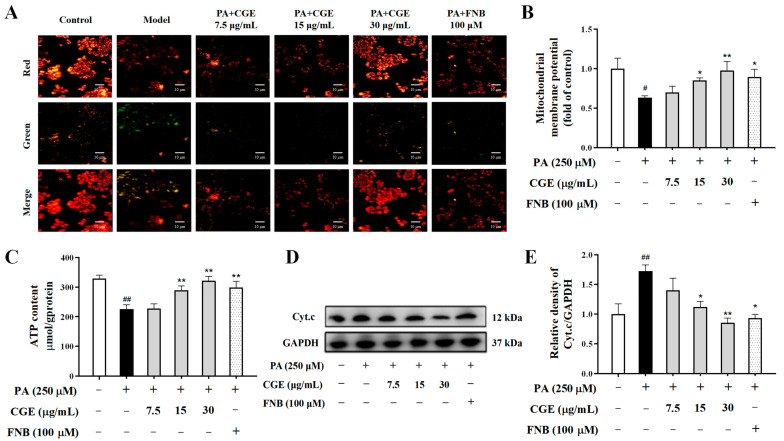
CGE maintained mitochondrial homeostasis in PA-induced HepG2 cells. (**A**) The mitochondrial membrane potential was observed using the JC-1 detection kit and fluorescence microscope, scale bar = 10 µm. (**B**) The relative red/green fluorescence intensity of JC-1 staining. (**C**) The ATP content was measured by a microplate reader. (**D**,**E**) The level of Cyt.c expression was determined by Western blot. Data are expressed as the mean ± SEM (*n* = 3), ^#^ *p* < 0.05, ^##^ *p* < 0.01 versus the untreated group; * *p* < 0.05, ** *p* < 0.01 versus the PA-only treated group.

Most oxidative stress occurs in intracellular mitochondria. Therefore, we observed the changes in mitochondrial homeostasis in PA-induced HepG2 cells. The red-green fluorescence ratio of HepG2 cells treated with PA was decreased compared with the normal group, indicating a significant decrease in intracellular mitochondrial membrane potential (Figure 4A,B). In addition, the intracellular ATP content was decreased, and the expression level of Cyt.c was significantly increased in PA-induced HepG2 cells (Figure 4C–E). CGE treatment restored the mitochondrial membrane potential, increased the ATP content, and downregulated the expression of Cyt.c. This suggests that CGE could effectively improve mitochondrial homeostasis in PA-induced HepG2 cells.

### 3.5. CGE Reduced Lipid Accumulation in PA-Induced HepG2 through PPARα Upregulation

**Figure 5:**
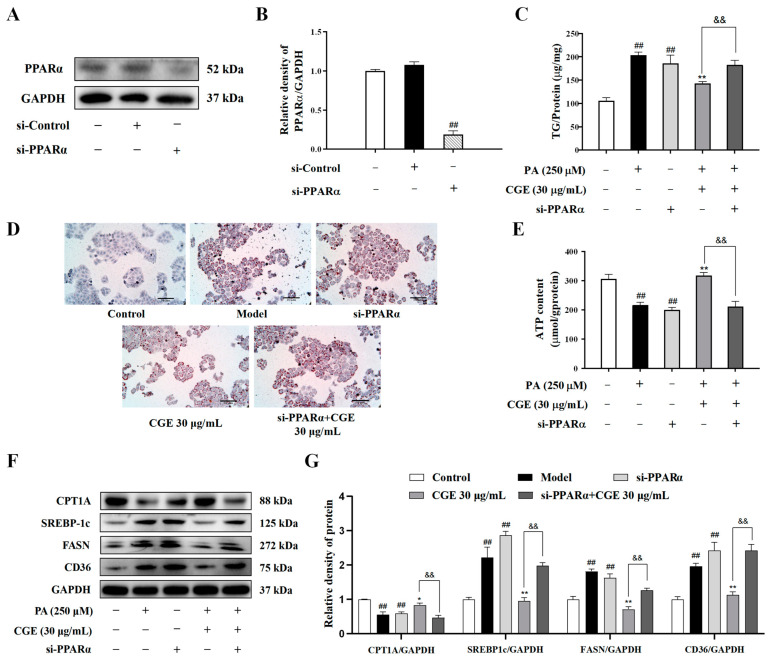
CGE reduced lipid accumulation in PA-induced HepG2 through PPARα upregulation. (**A**,**B**) Immunoblotting assay for PPARα expression. (**C**) The TG content was measured by the microplate reader. (**D**) Oil red O staining was analyzed by a microscope, scale bar = 10 µm. (**E**) The ATP content was measured by a microplate reader. (**F**,**G**) The expression of CPT1A, SREBP-1c, FASN, and CD36 protein were detected by Western blot. Data were expressed as the mean ± SEM (*n* = 3). ^##^ *p* < 0.01 vs. control group; * *p* < 0.05, ** *p* < 0.01 versus PA group; and ^&&^ *p* < 0.01 versus CGE group.

To further verify that CGE ameliorated the lipid metabolism disorder by regulating PPARα, we knocked down the endogenous PPARα gene using PPARα siRNA (Figure 5A,B). The effect of CGE on reducing the PA-induced TG content and lipid droplet quantity in HepG2 cells was reversed by si-PPARα (Figure 5C,D). Moreover, the si-PPARα treatment attenuated the effect of CGE on the increasing ATP content in PA-induced HepG2 cells (Figure 5E). Compared with the model group, CGE could upregulate CPT1A and downregulate the expressions of SREBP-1c, FASN, and CD36. After the interference of si-PPARα, the regulatory effect of CGE on the target protein related to lipid metabolism was significantly weakened (Figure 5F,G). These results suggest that CGE maintained fatty acid metabolic balance and reduced lipid accumulation in PA-induced HepG2 cells based on the upregulation of PPARα.

### 3.6. CGE Ameliorated Hepatic Steatosis in HFD-Fed MASLD Mice

**Figure 6:**
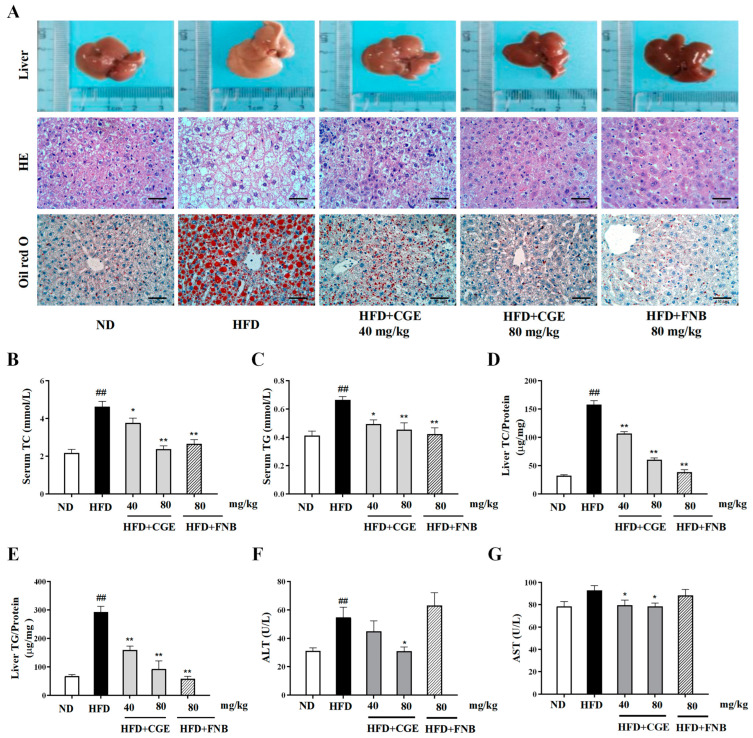
CGE ameliorated hepatic steatosis in HFD-fed MASLD mice. (**A**) Relevant images of liver appearance, oil red O, and H&E staining; scale bar = 50 µm. (**B**,**C**) The contents of serum TC and TG. (**D**,**E**) The TC and TG contents in liver tissue. (**F**,**G**) The contents of serum ALT and AST. Data are expressed as the mean ± SEM (*n* = 10). ^##^ *p* < 0.01 versus the ND group; * *p* < 0.05, ** *p* < 0.01 versus the HFD group.

To further observe the effect of CGE in vivo, the MASLD model was established by a 60% high-fat diet, and CGE was administered by gavage. MASLD mice are often accompanied by lipid accumulation and obesity. The body weight, liver weight, and liver index of mice in the HFD group increased, while those in the CGE group decreased significantly (Supplementary Figure S2A–C). Compared with the normal group, the liver of the HFD-fed mice was significantly enlarged and yellow. Compared with the HFD group, CGE treatment improved this situation to varying degrees. Through the HE staining of liver tissue sections, we found that the hepatocytes in the normal group were arranged in a cord-like structure with normal structure, while the HFD group showed diffuse lipid vacuoles and inflammatory infiltration around them. CGE treatment improved liver steatosis and inflammatory infiltration significantly. The results showed that red lipid droplets were significantly increased in the HFD group and decreased after CGE treatment (Figure 6A). Compared with the normal group, the TG and TC contents were significantly increased in the serum and liver tissue of the HFD group. However, CGE treatment reduced the contents of TG and TC (Figure 6B–E). HFD significantly increased serum AST and ALT levels compared with the normal group, while they were reduced after CGE treatment (Figure 6F,G). These results indicate that CGE could ameliorate hepatic steatosis and liver injury in HFD-induced MASLD mice.

### 3.7. CGE Improved Lipid Accumulation and Oxidative Stress in HFD-Fed MASLD Mice

**Figure 7:**
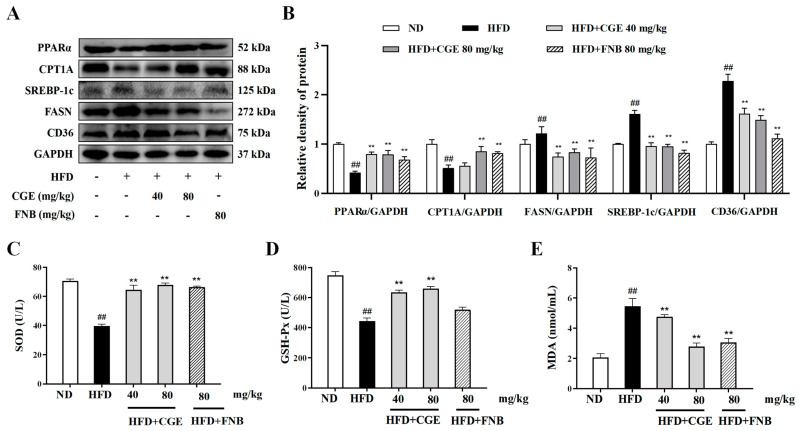
CGE improved lipid accumulation and oxidative stress in HFD-fed MASLD mice. (**A**,**B**) The levels of PPARα, CPT1A, SREBP-1c, FASN, and CD36 protein expression were determined by Western blot (*n* = 3). (**C**–**E**) The activities of GSH-Px, MDA, and SOD were measured by a microplate reader (*n* = 10). Data are expressed as the mean ± SEM, ^##^ *p* < 0.01 versus the ND group; ** *p* < 0.01 versus the HFD group.

To observe lipid metabolism in the liver of mice, we also detected hepatic PPARα and its related protein expression. Compared with the normal group, the expression levels of FAO-related proteins (PPARα and CPT1A) were significantly decreased, and the expression levels of fatty acids synthesis and transport-related proteins (SREBP-1c, FASN, and CD36) were significantly increased in the liver tissue of the HFD group. Compared with the HFD group, CGE treatment increased the PPARα and CPT1A protein and decreased the SREBP-1c, FASN, and CD36 protein in liver tissues (Figure 7A,B). The role of oxidative stress injury as a pivotal factor in the pathogenesis from MASLD to MASH cannot be overstated. The oxidative stress status of mice was observed by detecting the activities of antioxidant enzymes in mice. Compared with the normal group, HFD significantly reduced the activities of antioxidant enzymes SOD and GSH-Px and increased the lipid peroxide MDA content. Compared with the HFD group, CGE treatment significantly increased the activities of SOD and GSH-Px and decreased the lipid peroxide MDA content (Figure 7C–E). These results indicate that CGE ameliorated lipid metabolism disorders and oxidative stress in the HFD-induced MASLD mice.

### 3.8. Molecular Docking of Bioactive Compounds against PPARα Target

**Figure 8:**
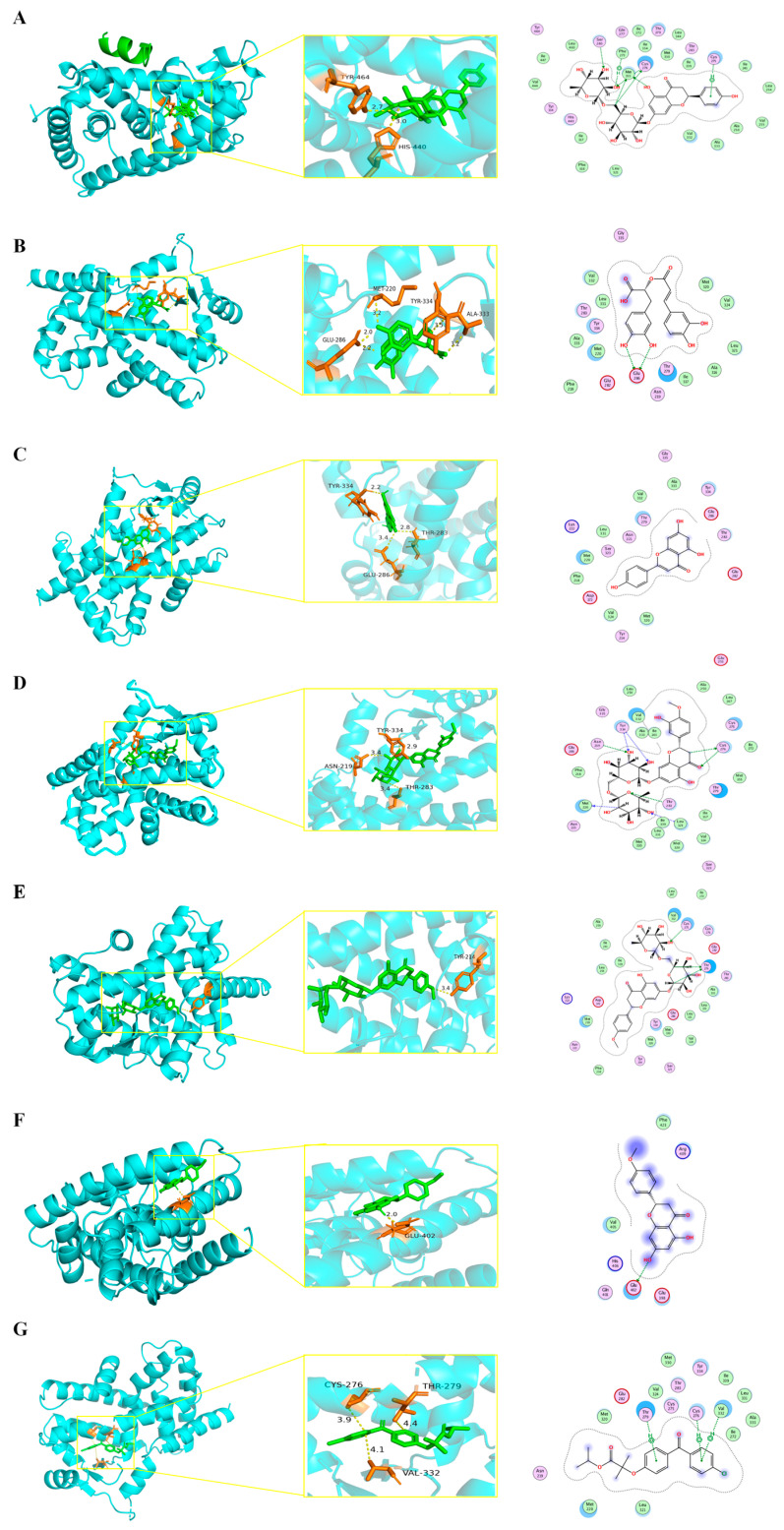
Molecular docking of active compounds from CGE against the PPARα target. Docking model of (**A**) narirutin, (**B**) rosmarinic acid, (**C**) apigenin, (**D**) hesperidin, (**E**) didymin, (**F**) isosakuranetin, and (**G**) fenofibrate against the PPARα target.

To evaluate the affinity of the active compounds for the PPARα target, we performed molecular docking analysis. As the results show in Figure 8, each active compound is bound to PPARα protein through visible hydrogen bonds. The binding energies of PPARα with narirutin, hesperidin, rosmarinic acid, didymin, or apigenin were lower than −5 kcal/mol. Remarkably, narirutin had the lowest binding energy of −9.83 kcal/mol with the PPARα target, indicating highly stable binding (Table 1).

## 4. Discussion

Modern diet and lifestyle aggravate the prevalence of obesity and diverse metabolic syndrome. MASLD is a chronic condition characterized by excess lipid accumulation in the hepatocytes resulting from an increased uptake in free fatty acids and excessive *de novo* lipogenesis. Researchers are still searching for alternative or complementary therapies because the medical management of MASLD is limited. Therefore, traditional Chinese herbs with multiple targets and pharmacological activities have great potential and development value. The results of the component analysis showed that CGE is rich in narirutin, rosmarinic acid, didymin, etc. All of these components have good effects on the prevention and treatment of MASLD. Citrus flavonoids contain rich narirutin, which can inhibit the expression of phosphorylated NF-κB and MAPKs to exert hepatoprotective and anti-inflammatory effects [28]. Rosmarinic acid can also reduce oxidative stress and regulate lipid metabolism disorders by increasing antioxidant enzyme activity and activating AMPK [29,30]. Didymin is a possible treatment because it reduces lipid deposition, inhibits inflammation, and promotes mitochondrial biogenesis [31,32]. Hesperetin improves MASLD by regulating hepatic metabolism and suppressing inflammation and oxidative stress [33,34]. The bioactive compounds derived from CGE have the potential to improve MASLD by reducing lipid accumulation, minimizing ROS production, and inhibiting inflammation. This further supports the notion that CGE could be a promising therapeutic candidate for treating MASLD.

A high-fat diet and dysregulation of lipid metabolism can lead to hepatic steatosis. Excessive lipid accumulation triggers a series of adverse consequences. The strategy for maintaining lipid metabolic homeostasis is to inhibit lipid synthesis and transport and enhance fatty acids β-oxidation. Despite the liver’s diligent efforts to rectify the imbalance caused by excessive lipid accumulation, the prolonged deposition of lipids ultimately results in impaired mitochondrial function. The decline in mitochondrial function not only exacerbates the disorder of lipid metabolism but also potentially induces intracellular oxidative stress [35]. We established the MASLD models induced by excess lipids in vitro and in vivo. In this study, CGE treatment promoted fatty acid oxidation (CPT1A) and lessened fatty acids synthesis and transport (SREBP-1c, FASN, and CD36) to decrease the intracellular fatty acid content. Notably, CGE treatment attenuated lipid accumulation in PA-induced HepG2 cells and MASLD mice. These regulatory effects depend on the upregulation of PPARα. Molecular docking results also showed that the main active components of CGE had low binding energy and highly stable binding with the PPARα target. In addition, it was reported that the significant effects of rosmarinic acid on MASH mice may be attributed to the activation of SIRT1/PPARα pathways [36]. Naringin increased fatty acid β-oxidation through regulating the expression of the PPARα protein [37]. Apigenin alleviated liver fibrosis by inhibiting p38/PPARα pathways in hepatic stellate cells [38]. This evidence further supports the fact that CGE promotes lipid metabolism by regulating PPARα activation, thereby alleviating MASLD.

Oxidative stress is mainly caused by lipid accumulation in MASLD. Fatty acids normally undergo β-oxidation reactions in mitochondria to produce energy. The excessive influx of fatty acids into mitochondria in hepatocytes can result in lipid peroxidation and an elevation in the production of ROS [39]. Under normal conditions, cells can effectively counteract physiological ROS formation through antioxidant defense systems. Unsaturated fatty acids undergo oxidation to produce a large amount of ROS and MDA. Such substances can diffuse from the starting position and further amplify oxidative stress damage [40]. Our results show the ROS and MDA contents were significantly increased, while SOD and GSH-Px activities decreased in HFD-fed mice and PA-treated HepG2 cells. However, CGE attenuated oxidative stress and enhanced the activity of antioxidant enzymes to mitigate hepatocyte damage.

At present, mitochondria are considered to play an important role in the onset of MASLD, which is also referred to as “mitochondrial disease” [41]. Excessive fat accumulation within the mitochondria can compromise mitochondrial function, which, in turn, affects cellular energy metabolism [42]. In MASLD, mitochondrial abnormalities in structure and function can reduce mitochondrial fatty acid β-oxidation and produce excessive ROS and lipid peroxides [43,44]. Mitochondrial membrane potential has an important effect on the metabolic activities of cells, such as regulating mitochondrial respiratory activity and the ATP synthesis rate. A PPARα agonist can ameliorate mitochondrial dysfunction and inhibit hepatic steatosis in MASLD mice [45]. Our results indicate that CGE potently inhibits a reduction in mitochondrial membrane potential and ATP synthesis in PA-treated HepG2 cells. Moreover, the role of CGE in promoting ATP synthesis and maintaining mitochondrial homeostasis in hepatocytes depends on the upregulation of PPARα. The results suggest that CGE could ameliorate the mitochondrial dysfunction resulting from lipid accumulation by regulating PPARα activation. A lower docking score also suggests that the binding affinity of active components of CGE for the PPARα protein exhibited greater potential. Furthermore, CGE also suppressed the release of Cyt.c from the inner mitochondrial membrane to the cytosol, thereby mitigating subsequent cellular apoptosis mediated by persistent mitochondrial damage.

Our findings revealed that CGE upregulated PPARα to reduce lipid accumulation without causing cytotoxicity. On the other hand, the presence of antioxidant effects and maintenance on mitochondrial homeostasis showed that CGE could be a promising choice for MASLD therapy. Our results give basic theoretical support for the use of CGE in the treatment of MASLD. Of course, this study has some limitations. Our experiment was based on the primary extract of *C. gracile*. However, we did not examine the protective effect of each component of CGE on the liver. In addition, the absorption, distribution, metabolism, and excretion of CGE in mammals need to be further studied. The contribution of CGE-derived metabolites and the breakdown of products to the hepatoprotective effect has not been clearly defined. These are worthy of further study in the future.

## 5. Conclusions

In summary, CGE exhibited a significantly ameliorative effect on MASLD by upregulating PPARα to improve lipid metabolism disorder. Moreover, CGE could attenuate oxidative stress and reduce mitochondrial oxidative damage to protect the liver from lipotoxicity. This study provides both theoretical and experimental foundations for the therapeutic application of CGE in MASLD.

## Figures and Tables

**Table 1:** Docking score results of active compounds from CGE for the PPARα target.

Compound	Docking Score (kcal/mol)
narirutin	−9.83
rosmarinic acid	−8.98
apigenin	−8.80
hesperidin	−8.00
didymin	−7.67
isosakuranetin	−2.90
fenofibrate	−7.60

## Data Availability

Data are available on request from the authors.

## Supplementary Materials

The following supporting information can be downloaded at: https://www.mdpi.com/article/10.3390/antiox13091136/s1, Figure S1: Effect of CGE and FNB on HepG2 cells viability; Figure S2: Effects of CGE on weight and liver weight of HFD-induced mice.
