# Supplementary material for: Clinopodium gracile Alleviates Metabolic Dysfunction-Associated Steatotic Liver Disease by Upregulating Peroxisome Proliferator-Activated Receptor α and Inhibiting Mitochondrial Oxidative Damage
Source: Antioxidants (Basel). 2024 Sep 20;13(9):1136. doi: 10.3390/antiox13091136 (PMC11428588; doi:10.3390/antiox13091136)
Supplement: Supplementary file 1 [file antioxidants-13-01136-s001.zip › antioxidants-3176544-supplementary.pdf]

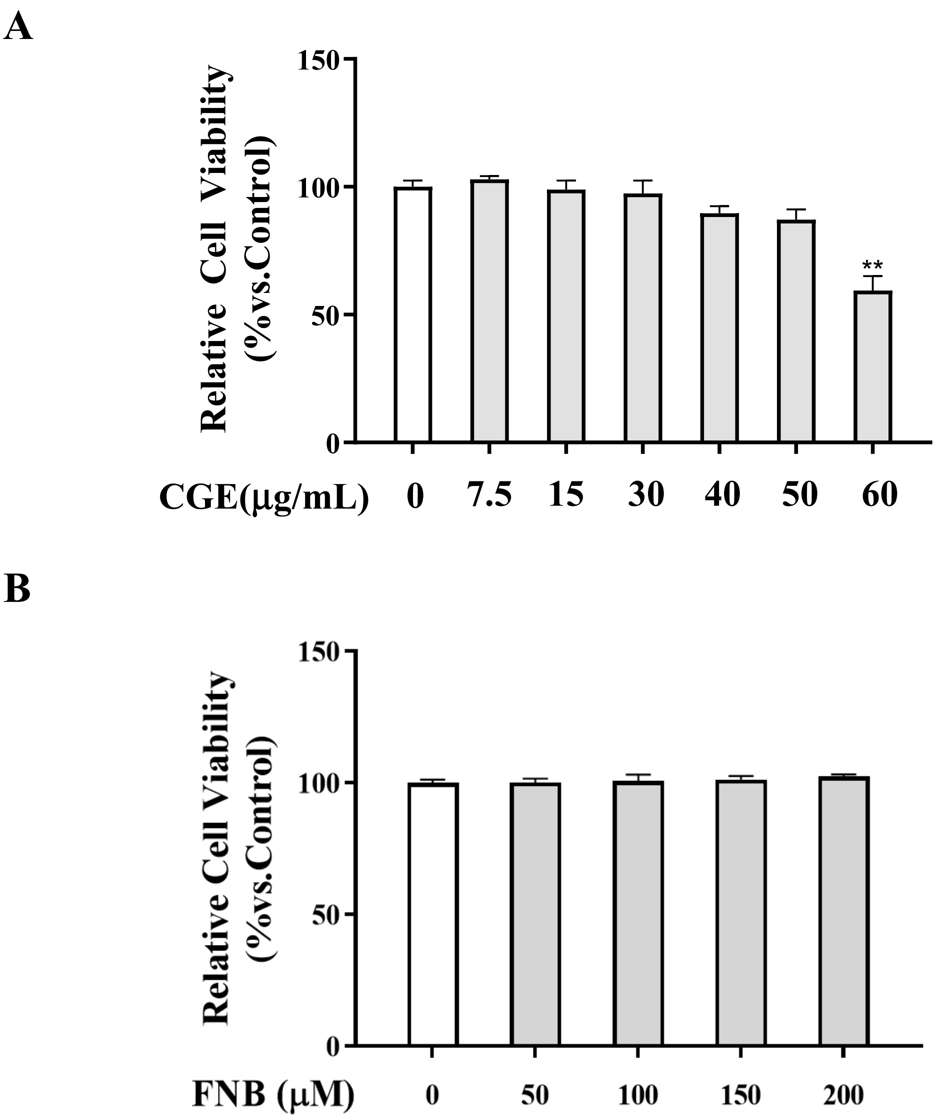

Figure S1. Effect of CGE and FNB on HepG2 cells viability. Cell viability after treatment with different concentrations of **(A)** CGE and **(B)** FNB. Data are presented as the mean ± SEM (n = 3), $^{*}P < 0.05$, $^{**}P < 0.01$ vs. untreated group.

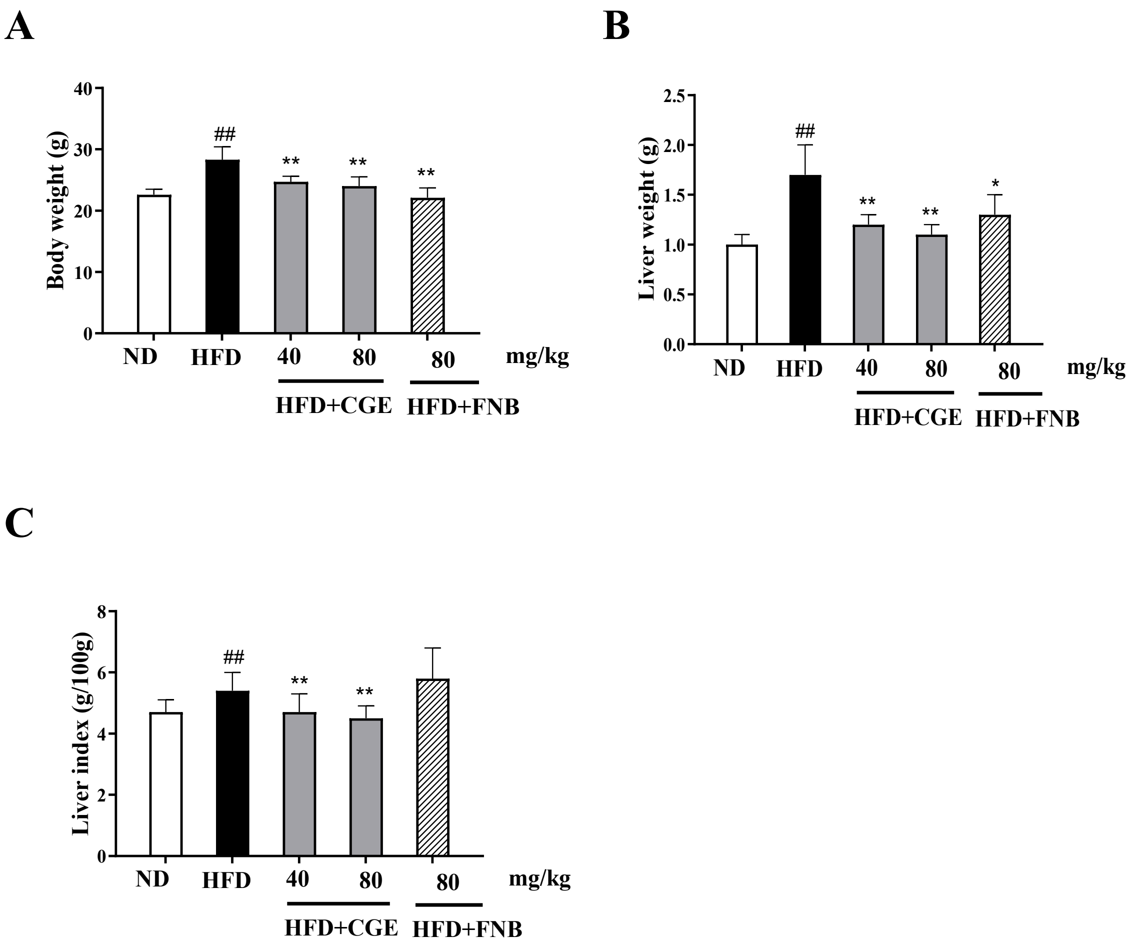

Figure S2. Effects of CGE on weight and liver weight of HFD-induced mice. **(A)** Body weight, **(B)** liver weight, and **(C)** liver index in HFD-induced mice. Data are presented as the mean ± SEM (n = 10), $^{\#}P < 0.05$, $^{\#\#}P < 0.01$ vs. the ND group; $^{*}P < 0.05$, $^{**}P < 0.01$ vs. the HFD group.
